# Sex differences in the relationship between pain and autonomic outflow during a cold pressor test

**DOI:** 10.1186/s13293-025-00743-2

**Published:** 2025-08-06

**Authors:** Laila A. Chaudhry, Yasmine Coovadia, Brittany K. Schwende, Danielle E. Berbrier, Will Huckins, Jinan Saboune, Derek A. Skolnik, Emily K. Van Berkel, Jeffrey S. Mogil, Charlotte W. Usselman

**Affiliations:** 1 Alan Edwards Centre for Research on Pain, McGill University, 3755 University Ave, Montreal, QC H3A 2B4 Canada; 2 Department of Psychology, McGill University, Montreal, Quebec Canada; 3 Department of Anesthesia, Faculty of Dental Medicine, McGill University, Montreal, Quebec Canada; 4 Cardiovascular Health and Autonomic Regulation Lab, Department of Kinesiology & Physical Education, McGill University, Montreal, QC Canada

**Keywords:** Cold pressor test, Tonic pain, Muscle sympathetic nerve activity, Autonomic nervous system, Sex differences

## Abstract

**Background:**

Chronic pain is partly maintained by the sympathetic nervous system, whose activity is best measured by muscle sympathetic nerve activity (MSNA). MSNA responses to acute pain have been thoroughly investigated, whereas MSNA responses to longer-lasting pain are poorly understood. Therefore, this study examined the relationship between pain ratings and peroneal MSNA during a tonic cold pressor test (CPT) in male and female participants.

**Methods:**

We obtained MSNA measures during a 6 min CPT in 18 young adult (20–33 years) men and women. Verbal pain ratings (0–10) and autonomic outcomes (heart rate [HR], mean arterial blood pressure [MAP], and MSNA) were assessed simultaneously at multiple time points across the CPT.

**Results:**

Pain, HR, and MAP increased in the initial 30s in both sexes. Females increased their MSNA burst frequency (BF) to a greater extent than males. Across the full CPT we observed a positive relationship between pain and HR in males, a positive relationship between pain and MSNA BF in females, and a negative relationship between pain and MSNA burst amplitude in females.

**Conclusions:**

Overall, males displayed a strong relationship between tonic pain and HR, an index of parasympathetic activity, whereas females displayed strong and offsetting relationships between tonic pain and purely sympathetic MSNA variables. These observations suggest sex differences in autonomic mechanisms during tonic pain, which may have relevance to ongoing efforts to modulate pain via manipulations of the autonomic nervous system, as well as sex/gender disparities in chronic pain prevalence.

**Supplementary Information:**

The online version contains supplementary material available at 10.1186/s13293-025-00743-2.

## Abstract (plain language summary)

Chronic pain, or pain lasting longer than 3 months, is partly maintained by the sympathetic nervous system, the system that initiates “fight or flight”-related stress. The activity of this system is best measured by muscle sympathetic nerve activity (MSNA), nerve impulses that act on blood vessels. MSNA responses to short-lasting pain have been thoroughly investigated, but MSNA responses to longer-lasting pain are poorly understood. Therefore, this study examined the relationship between pain ratings and MSNA during a long-lasting (6-min) cold-water immersion test in male and female participants. Verbal pain ratings (0–10), cardiovascular outcomes (heart rate, blood pressure), and MSNA were assessed simultaneously at multiple time points. Pain, heart rate, and blood pressure increased in the initial 30 s in both sexes, with females increasing their MSNA more than males. We observed that males displayed a strong relationship between pain and heart rate, whereas females displayed strong relationships between pain and MSNA variables. These observations suggest sex differences in nervous system responses during long-lasting pain, which may have relevance to sex/gender disparities in chronic pain populations.

**Supplementary Information:**

The online version contains supplementary material available at 10.1186/s13293-025-00743-2.

## Highlights

- In response to a prolonged cold pressor test, females showed greater pain-related sympathetic responses than males.
- Autonomic responses to tonic pain could have implications for chronic pain, and sex/gender differences in these responses could help to explain sex/gender disparities in chronic pain populations.

**Supplementary Information:**

The online version contains supplementary material available at 10.1186/s13293-025-00743-2.

## Introduction

Chronic pain is often comorbid with other conditions, particularly cardiovascular diseases [1, 2], leading to increased rates of cardiovascular mortality in pain patients [3–5]. Patients who develop chronic postsurgical pain have almost twice the prevalence of hypertension than patients who do not [6], and chronic pain patients exhibit reduced heart rate variability [7–11], a marker for cardiovascular disease risk [12]. Brain regions associated with autonomic control of the cardiovascular system overlap substantially with those related to nociceptive processing [13], which may account for the link between chronic pain conditions and dysregulation in the autonomic—and, in particular, sympathetic—control of the cardiovascular system [3, 14, 15].

Sympathetic nervous system activation can be inferred from non-invasive cardiovascular parameters (e.g., blood pressure) but the gold-standard method for quantifying sympathetic outflow in humans is microneurography [16, 17]. Microneurography is a neurophysiological technique in which a conductive microelectrode is inserted into a peripheral nerve of an awake human participant in order to record and visualize the traffic of nerve impulses [18]. Muscle sympathetic nerve activity (MSNA) can be measured via the microneurographic targeting and recording of efferent postganglionic sympathetic nerve fibers innervating vascular smooth muscle, and serves as a direct measure of purely sympathetic—as opposed to parasympathetic—activity. MSNA is commonly quantified in cardiovascular research due to its role in homeostatic blood pressure regulation (e.g., baroreflex) mechanisms [19]. However, microneurographic recordings of MSNA have also been applied within the pain research field to directly quantify sympathetic responses to noxious stimuli and better understand relationships between sympathetic outflow and pain [20–25].

In general, MSNA increases when a participant is exposed to a noxious stimulus. This relationship has been demonstrated using various and diverse pain assays, including: soap solution in the eye [22], nailbed mechanical pressure [22], mechanical skin pressure [23], and the cold pressor test (CPT) in which a participant’s hand is immersed in a cold water bath typically for 0.5–3 min [24, 26]. What remains poorly understood are MSNA responses to sustained (i.e., tonic or chronic) pain. Individuals with cluster headaches demonstrate higher levels of basal MSNA [27]. Moreover, Fazalbhoy and colleagues [20, 28] observed that a model of tonic visceral pain (i.e., bolus intramuscular injection of hypertonic saline, associated with pain lasting ~ 60 min) resulted in heterogeneous MSNA responses, with some participants progressively increasing and others progressively decreasing MSNA over the duration of the stimulus. Neither study included enough female participants to assess whether sex contributed to this variability, nor were sexdisaggregated data reported in the existing CPT studies [24, 26]. Thus, the effects of tonic pain on MSNA remain to be firmly established in both sexes.

As experimental models of tonic pain—particularly CPT which induces a deep, tonic, aching pain—have greater clinical relevance for chronic pain than do acute pain models [29–31], the primary aim of this study was to explore interactions between time, pain, and indices of autonomic activity, including MSNA, across a tonic pain stimulus (i.e., a 6 min-long CPT). We hypothesized that MSNA and pain would steadily and concomitantly increase throughout the CPT. Additionally, given the large body of evidence supporting sex and/or gender differences in pain perception [32–34] and blood pressure regulation [35], our secondary aim was to examine the effect of sex/gender on changes in pain and MSNA.

## Materials and methods

### Sample size and participants

Sample size calculations were based off of an existing pain and MSNA study [36] which examined pain responses to CPT in two groups MSNA responders (*n* = 12) and non-responders (*n* = 12); a sensitivity calculation for this study yielded an effect size of 0.48. Using this effect size, a type 1 error of 0.05, and a desired power of 0.8, a sample size of *n* = 9 per group was calculated for a mixed model RM ANOVA (G*Power 3.1; Dusseldorf, Germany).

We tested young (18–35 years), healthy men (*n* = 9) and women (*n* = 12) free from cardiovascular, respiratory, endocrinological, and chronic pain disorders (see Table 1). We also excluded cigarette smokers. Three female participants were removed due to low-quality MSNA data, leaving 9 female participants. Although potential effects of sex and gender on our outcomes are likely intertwined [33, 37], this study was not designed to assess the effects of sex and gender separately. Rather, we sought to analyze the effect of sex by dividing outcomes based on sex assigned at birth (male/female), and therefore the term ‘sex difference’ is used throughout. However, we note that our intake questionnaires did assess gender identity, and that all participants self-reported as cisgender or did not report at all.

**Table 1:** Participant demographics

	Females (*n* = 9)	Males (*n* = 9)	*P*
**Age** **(Mean ± SD)**	25 ± 3.6	22.7 ± 3.0	0.16
**Race**			
White	5	5	
Asian	1	1	
Mixed Race	1	1	
Unspecified	2	2	
**BMI**	21 ± 1.2	24.3 ± 2.4	< 0.01
**Physical Activity** **(hours/week)**	3.8 ± 2.8	4.8 ± 6.5	0.67
**Prescription Medications**	sertraline (*n* = 1), spironolactone (*n* = 1)	-	

### Experimental design

Women were tested during the early follicular phase of the menstrual cycle (days 1–5; day being the first day of menses; *n* = 2) or the low-hormone placebo phase of hormonal contraceptive use (*n* = 7). All participants were tested at the same time of day (08:00 ± 1 h) to minimize any effects of circadian variations on resting MSNA. During a separate visit to the lab prior to testing, participants were familiarized with all non-invasive aspects of testing (see below), including an abbreviated CPT. Participants were coached to remain physically relaxed and to not hold their breath throughout the CPT in order to minimize movement- and respiration-induced alterations in the MSNA signal during testing [38, 39].

On the test day, all participants arrived at the laboratory having fasted a minimum of 3 h, and having abstained from caffeine, strenuous exercise, alcohol, and analgesics for 12 h. On arrival, participants were instructed to void their bladders. Participants were then positioned supine on a padded table for instrumentation. After ~ 15 min of stable supine rest, manual sphygmomanometry was used to obtain three manual blood pressure (BP) values that were used to calibrate finger photoplethysmography values. Microneurography was then conducted to obtain the MSNA signal. Following attainment of an adequate MSNA site, 10 min of quiet rest were recorded to quantify baseline values of all outcomes. A 6-min CPT was then performed, consisting of placement of the participant’s hand up to their wrist in ice water (∼4 °C). This is an extended version of a well-established CPT protocol which elicits both pain and sympathetic reactivity in humans [e.g., 40, 41–46]; our pilot work indicated that 6-min was the upper limit that could be tolerated by most participants. All participants tolerated 6 min of CPT, and reported pain ratings verbally on a numerical rating scale (0–10) at rest and at four predetermined time points over the course of the CPT (time points: 30 s, 2 min, 4 min, and 5 min 30 s).

### Instrumentation

Heart rate (HR) was measured using a standard 3-lead electrocardiogram (ECG). BP was measured on a beat-by-beat basis using finger photoplethysmography (Finometer MIDI, Finapres, Amsterdam, Netherlands), which involved the placement of a small cuff around the participant’s third or fourth finger on the hand contralateral to the CPT. HR and continuous BP signals were sampled continuously at a frequency of 1.0 kHz and saved for offline analysis (PowerLab and LabChart v8, ADInstruments). Microneurography was used to record multiunit postganglionic MSNA from the common peroneal nerve (NeuroAmp EX, ADInstruments) [18]. Briefly, an insulated tungsten recording electrode (35 mm in length, 200 μm in diameter, 2 ± 0.4 MΩ impedance) was inserted transcutaneously into the peroneal nerve, and a reference electrode was inserted subcutaneously 1–3 cm away from the recording site. An adequate MSNA signal consisted of pulse-synchronous bursts of activity that increased in firing frequency during voluntary apnea and remained unchanged during arousal to a loud noise [47]. The raw sympathetic signal was amplified 100× by a head-stage and the total amplification was 20,000×. The signal was then band-pass filtered (700–2000 Hz), full wave rectified, and integrated (time constant 0.1 s). Sympathetic activity was recorded at 10.0 kHz.

### Data analyses

ECG and calibrated BP waveforms were analyzed to determine HR and mean arterial blood pressure (MAP), respectively. Bursts of MSNA were detected using a semi-automated peak detection algorithm (LabChart V8) based on a 3:1 signal-to-noise ratio and confirmed by a trained microneurographer following a shift of the MSNA signal to account for the neural conduction latency within each subject, aligning each sympathetic burst with the cardiac cycle that initiated it [18]. Bursts of sympathetic activity were quantified as burst frequency (BF; number of bursts/min) and burst amplitude (BA; percentage of peak baseline voltage). Both are standard measures used to quantify MSNA: MSNA BF informs about sympathetic neuronal firing rates, and MSNA BA about axon size of recruited sympathetic neurons and resulting neurotransmitter release [48]. Finally, they can be multiplied together to calculate total MSNA, which functions as a measure of the combined effects of neural firing rates and neurotransmitter release on a blood vessel.

Statistical analyses were performed, and figures created using GraphPad Prism v.9 (La Jolla, CA). Shapiro Wilk tests for normality were conducted for all variables at each time point. Grubbs’ test was used to identify outliers within each variable and analysis. All data were expressed as means ± standard error (SEM), and α was set at < 0.05 to establish statistical significance.

**Fig. 1:**
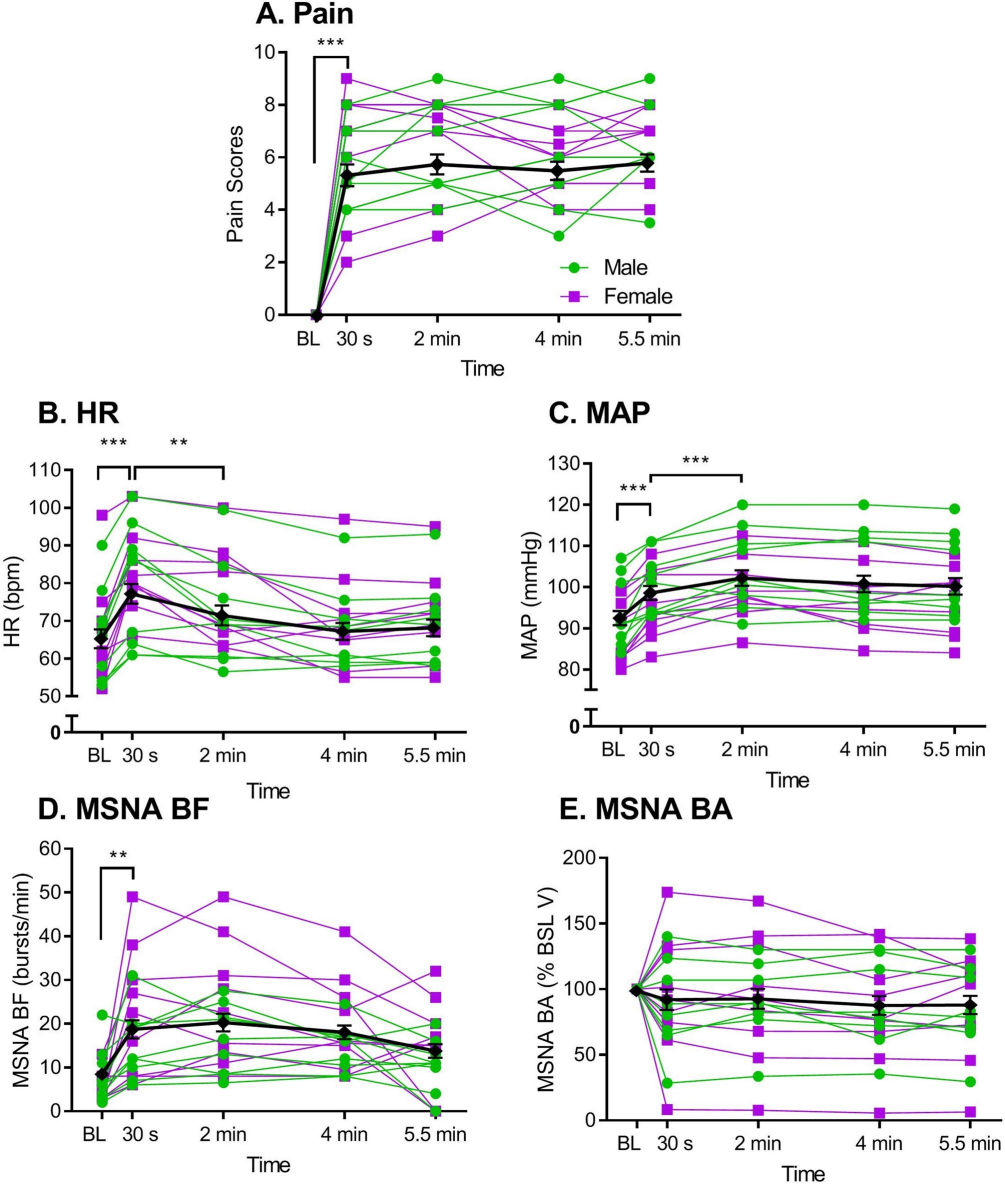
Time course of pain (**A**) and autonomic variables (**B**, heart rate [HR]; **C**, mean arterial pressure [MAP]; **D**, muscle sympathetic nerve activity burst frequency [MSNA BF]; and **E**, muscle sympathetic nerve activity burst amplitude [MSNA BA]) before (i.e., at baseline [BL]) and at various time points during a CPT. Black symbols indicate mean ± SEM; purple symbols represent female participants (*n* = 9) and green symbols represent male participants (*n* = 9; *n* = 8 for MSNA BA). **p < 0.01, ***p < 0.001 as indicated

Baseline (BL) values of all outcomes were extracted as the average of 10 min of quiet rest prior to the CPT. To assess absolute levels of pain, HR, MAP, and MSNA (BF, BA, and total MSNA), we extracted the average of the 60-s period centered around each pain rating time point (i.e., 0 to 60 s, 90 to 150 s, 210 to 270 s, 300 to 360 s). Two-way repeated measures analyses of variance (ANOVAs; sex x time) were performed. Where main effects were significant, a *post-hoc* Sidak multiple comparisons test was used determine how variables changed throughout the duration of the CPT within each sex (Fig. 1).

**Fig. 2:**
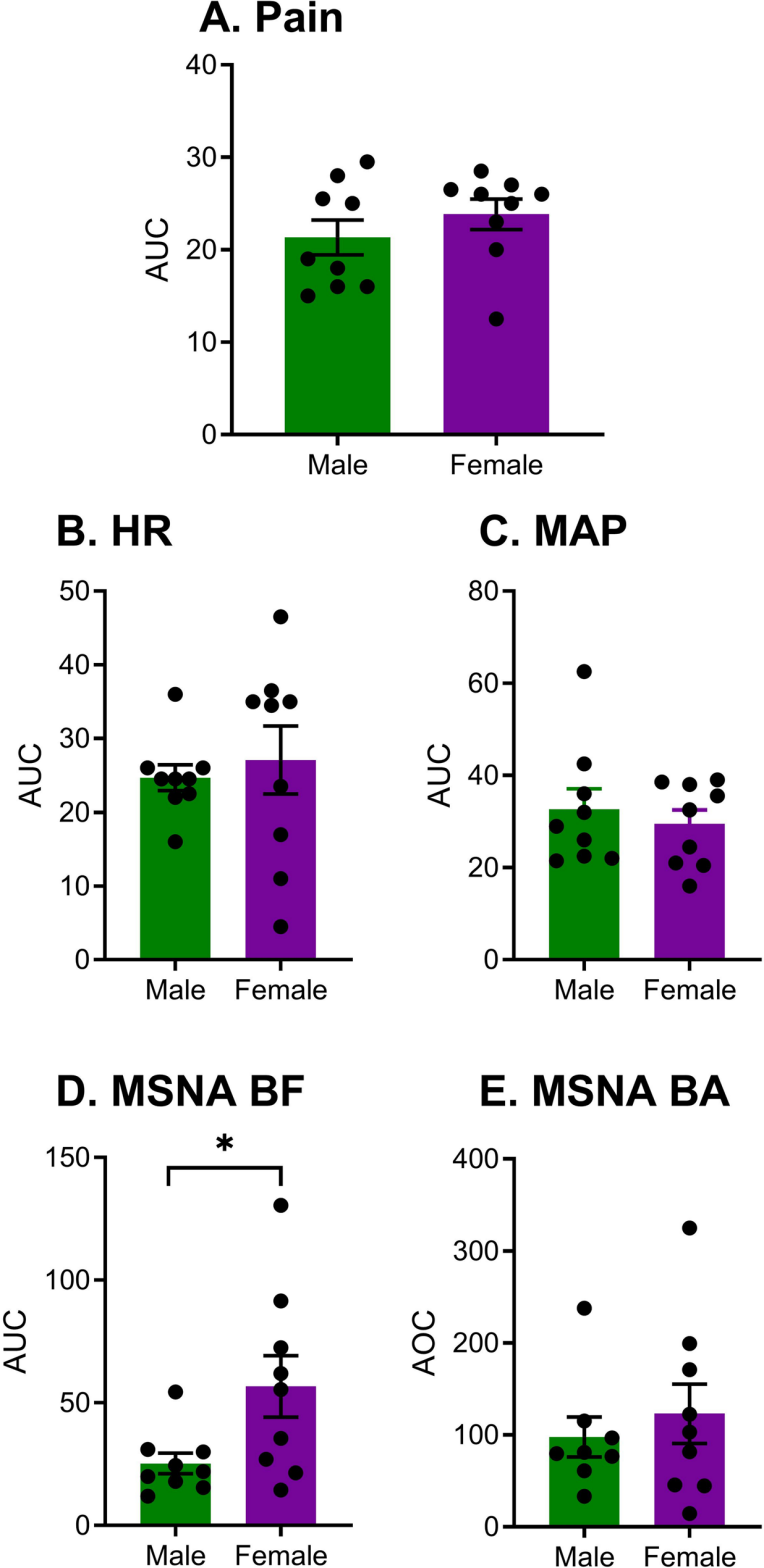
Areas-under-the-curve (AUC; in A–D) or areas-over-the-curve (AOC; in E) of pain (**A**) and autonomic responses (**B**, heart rate [HR]; **C**, mean arterial pressure [MAP]; **D**, muscle sympathetic nerve activity burst frequency [MSNA BF]; and **E**, muscle sympathetic nerve activity burst amplitude [MSNA BA]) to the CPT expressed as relative changes from baseline and stratified by sex. Bars represent mean ± SEM. *p < 0.05 as indicated

To assess cumulative effects of changes over time in pain, HR, MAP, and MSNA, we calibrated our variable values relative to baseline, and then calculated the area-under-the curve (AUC) of these baseline-relative values for pain, HR, MAP, and MSNA BF using the trapezoidal method. For MSNA BA, where means decreased from baseline, absolute values, or area-over-the-curve (AOC), was used instead. Unpaired Student’s *t*-tests were used to compare AUC between the sexes (Fig. 2).

**Fig. 3:**
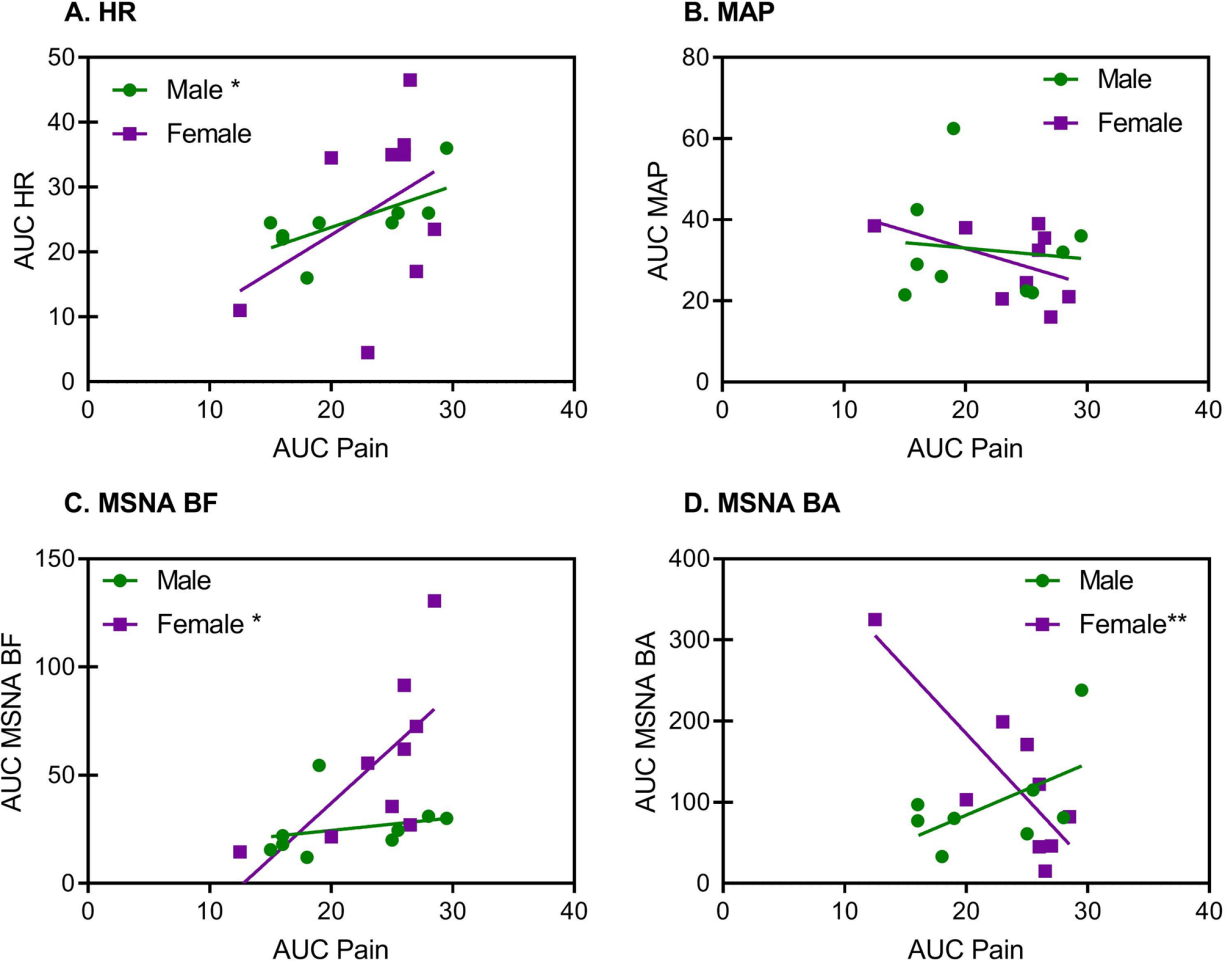
Sex-dependent relationships between pain (AUC) and autonomic variables (**A**, heart rate [HR]; **B**, mean arterial pressure [MAP]; **C**, muscle sympathetic nerve activity burst frequency [MSNA BF]; and **D**, muscle sympathetic nerve activity burst amplitude [MSNA BA]). Lines represent linear regressions. *p < 0.05, **p < 0.01 within-sex

To assess whether overall changes in HR, MAP, and MSNA were associated with overall changes in pain and the strength of their relationships, we calculated linear regressions and Pearson correlations of AUCs of full CPT (baseline to 5.5-min) HR, MAP, and MSNA responses. We then conducted unpaired F-tests of slopes to assess if these relationships differed between the sexes (Fig. 3).

## Results

All participants reported no pain (0) at baseline. When expressed relative to baseline, all variables were normally distributed as determined by Shapiro-Wilk tests. Using Grubbs’ test for outliers, one male participant’s data was removed from all MSNA BA analyses.

### Absolute levels across time course

Repeated-measures ANOVA revealed a main effect of time for pain (*F*_4,64_ = 113.1, *p* < 0.001; η^2^ = 0.73; Fig. 1A), HR (*F*_4,64_ = 24.9, *p* < 0.001; η^2^ = 0.14; Fig. 1B), MAP (*F*_4,64_ = 46.0, *p* < 0.001; η^2^ = 0.16; Fig. 1C), and MSNA BF (*F*_4,64_ = 10.4, *p* < 0.001; η^2^ = 0.22; Fig. 1D). There was no main effect of time observed for MSNA BA (*p* = 0.23; Fig. 1E). There were no main effects of sex, nor any time-by-sex interactions, on any of these variables (0.16 < *p* < 0.94).

Pain (*p* < 0.001; Fig. 1A), HR (*p <* 0.001; Fig. 1B), MAP (*p* < 0.001; Fig. 1C) and MSNA BF (*p* < 0.001; Fig. 1D) all increased significantly from baseline to 30-s. Both HR (*p* = 0.003) and MAP (*p =* 0.001) increased further from the 30-s to the 2-min time point.

### Areas under/over the curve

Although there were no significant time-by-sex interactions observed at any particular time point in absolute measures of pain, HR, MAP, or MSNA, we wanted to understand how the sexes may have differed in their overall CPT responses expressed relative to baseline over the entire 5.5 min time period. We thus calculated areas under the curve for each variable. There was no significant sex difference in pain (*t*_16_ = 1.0, *p* = 0.33; Fig. 2A), HR (*t*_16_ = 0.48, *p* = 0.63; Fig. 2B) or MAP AUC (*t*_16_ = 0.59, *p* = 0.56; Fig. 2C). With respect to measures of MSNA, we observed a significant sex difference in MSNA BF AUC (*t*_16_ = 2.4, *p =* 0.03; η^2^ = 0.26; Fig. 2D) but not MSNA BA (*t*_15_ = 0.64, *p =* 0.53; Fig. 2E). That is, over the full testing period, female participants displayed 2.2fold higher changes in MSNA BF than male participants. We also calculated total MSNA (BF x BA), which was not significantly different between male and female participants (see Supplementary Fig. 1).

### Correlations of autonomic indices with pain

Males displayed a significant positive relationship between pain and HR (*r* = 0.69, *p* = 0.04; Fig. 3A) whereas females showed a positive but non-significant relationship between pain and HR (*r* = 0.41, *p* = 0.27; Fig. 3A). However, regression slopes were not significantly different between sexes (*p* = 0.59). Pain and MAP were not related in males or females (*r*= -0.11 and *r*= -0.49 respectively; Fig. 3B). Females showed a significant positive relationship between pain and MSNA BF (*r* = 0.67, *p* = 0.048; Fig. 3C), whereas males did not (*r* = 0.26, *p* = 0.49; Fig. 3C). The slopes of the male and female regression lines only approached significance (*p* = 0.06). Finally, females displayed a significant negative relationship between pain and MSNA BA (*r*= -0.82, *p* = 0.007; Fig. 3D), whereas males showed a positive but non-significant relationship (*r* = 0.57, *p* = 0.14; Fig. 3D). Regression slopes for MSNA BA were significantly different by sex (*F*_1,13_ = 15.6, *p* = 0.002).

## Discussion

Despite the well-established positive linear relationship between acute pain and MSNA, our findings indicate that a tonic painful stimulus maintains a complex relationship with MSNA variables—as well as other autonomic indices like BP and HR—over time. Despite the difficulties in obtaining high-quality MSNA signals over a 6 min CPT, we were able to obtain such data in enough participants to examine sex differences during tonic pain for the first time and observed that autonomic responses to pain change in a sexdependent manner.

### Pain and the autonomic nervous system

As expected, the noxious stimulus significantly increased pain levels as well as HR, BP, and MSNA BF (see Fig. 1). The brain regions associated with control of the autonomic system are known to overlap substantially with those related to nociception [13], and thus it is not surprising that CPT-induced activation of this system led to parallel initial increases in both pain perception and autonomic variables. Although variability was noted both between participants and over time within participants, after the initial increase, mean responses for most variables stayed fairly constant over the entire time course of the CPT. The similar time courses of pain scores and the autonomic variables over time are likely due to the homeostatic relationship between pain and stress, in which acute pain can produce acute stress [49], and acute stress can inhibit pain (via stress-induced analgesia; [50]). This relationship may be mediated by a baroreceptor feedback loop, such that: (1) pain increases sympathetic arousal via a somatosensory reflex, thereby increasing BP; (2) increased BP stimulates baroreceptors, which trigger descending pain inhibition [15]; and, (3) pain inhibition returns arousal levels to a state of homeostasis [51, 52]. Thus, this homeostatic loop likely involves the autonomic as well as the sensory nervous systems, with the nucleus tractus solitarius (NTS) serving as the interface between them. The NTS receives significant afferent input from the nociceptive spinal cord laminae and the vagus nerve, which regulates the baroreflex [13, 15]. The two efferent arms of the baroreflex, of course, are the parasympathetic and sympathetic nervous systems.

### Effects of sex on the contribution of the parasympathetic system to pain

In the first 30 s, pain, HR, and BP significantly increased in both sexes (see Fig. 1). Additionally, males displayed a significant positive relationship between HR and pain throughout the CPT (see Fig. 3A), but little relationship with purely sympathetic variables. It is not uncommon for these variables to show dissociated relationships [53–55]. Given that HR and BP are both influenced by cardiovagal/parasympathetic outflow [56], which maintains a generally inverse relationship with HR, we suggest that pain, at least in males, may be influenced by parasympathetic withdrawal. In fact, a meta-analysis by Tracy and colleagues [57] showed that heart rate variability and parasympathetic outflow were disrupted in mixed-sex chronic pain patients, indicating that parasympathetic inhibition may be involved in the mediation of chronic pain as well.

Although females showed a moderate positive (but non-significant) correlation between HR and pain, they displayed much stronger relationships between pain and MSNA—a purely sympathetic variable (see below). These relationships suggest that physiological mechanisms involved in regulating pain initiation and maintenance differ between the sexes.

### Effects of sex on the contribution of the sympathetic system to pain

Across the CPT as a whole, females displayed greater MSNA BF to the CPT than did males (see Fig. 2D). It is fairly well established that young males have higher MSNA BF at rest than young females [58], which we observed as well (male: 8.2 ± 2.1; female: 5.3 ± 1.2), but sex differences response to stressors including the CPT remain an area of ongoing investigation. Existing studies investigating the MSNA response to CPT have reported no differences between sexes [59–62]. It is important to note, however, that the duration of the CPT in these studies were shorter than in the present study (i.e., 2-min [59, 60] and 3-min [61, 62]). It is possible that more prolonged cold pain may reveal sex differences in adaptation of cold afferents [63] or thermoregulatory mechanisms [64, 65] that could lead to the sex differences in cumulative MSNA BF observed here. It would be worthwhile to repeat this experiment using a different pain modality (e.g., hypertonic saline) to see whether the sex differences in MSNA BF relate to cold pain specifically or pain more generally.

In addition to changes in MSNA BF in females, we observed a significant positive relationship between pain and MSNA BF in females but not males, as well as a significant negative relationship between pain and MSNA BA in females but not males (Fig. 3C and D). Taken together, we can conclude that females have strong sympathetic reactions in response to, or in association with, tonic pain. It is understood that BA and BF function independently to some extent, dependent on the type of stimulus applied [66–70]. Indeed, sex [71, 72] and pain [73] have also been shown to differentially affect these two MSNA measures. Moreover, opposing relationships observed between BF and BA may be explained as follows. BF and BA may act in mutual counterbalance as a homeostatic mechanism to maintain total MSNA levels (see Supplementary Fig. 1), and thereby BP, which was mostly stable throughout the CPT in females (see Fig. 1C). MSNA BF is a measure of neuronal firing rates and may be increased in females in order to dampen pain perception via the initiation of stress-induced analgesia. We speculate that MSNA BA, as the other variable making up total MSNA, is correspondingly reduced in order to ensure total MSNA outflow and resulting BP remain steady. Alternatively, because females’ BA was negatively associated with pain, it is possible that the CPT leads to neural recruitment of smaller sympathetic neurons (i.e., decreased recruitment of larger axons) such that each MSNA burst releases less neurotransmitter. Indeed, neuronal size, and thus BA, can be regulated independently from BF [48, 74], and smaller-diameter neurons likely release less neurotransmitter [75]. As such, these smaller neurons may in turn increase their BF to compensate, leading to the positive relationship with pain in females. If these smaller sympathetic neurons continue to be persistently activated alongside pain, the resultant increase in BF could sensitize spinal nociceptive pathways, overpower a depleted pain-inhibitory system, and lead to hyperalgesia [76]. Reversal of the normal negative pain-stress homeostatic feedback loop into a positive one has been observed in orofacial pain patients [77] and chronic low back pain sufferers [78] and can ultimately contribute to the development of chronic dysfunctional pain [15, 79].

### Strengths and limitations

To our knowledge, this is the first study to examine relationships between repeated measurements of both tonic pain and MSNA in both sexes. We note that certain statistical tests evinced suggestive but non-significant correlations that may have been affected by the limited sample size and a large amount of interindividual variability within the sample; we were able to recruit and obtain highquality MSNA data in 18 participants, which is considered a relatively large sample size for microneurography studies. Furthermore, additional data collection in the form of more frequent pain assessments would have allowed for more granularity in time-course analyses.

## Conclusions

In summary, we find a dynamic relationship between pain and autonomic indices, a relationship that appears to vary between the sexes. These data suggest that pain in males may be regulated by mechanisms primarily associated with the parasympathetic system, whereas pain in females may be more closely tied to sympathetic mechanisms. Despite this physiological explanation, it is of course possible that sociocultural (gender) factors may play a role and/or amplify the differences we observed. Given that chronic pain has been associated with chronic activation of the sympathetic nervous system [80, 81], and is more prevalent in women, we suggest that treatment strategies involving manipulations of the autonomic nervous system (e.g., neuromodulation) should pay special attention to sex and gender factors.

## Supplementary Information

Below is the link to the electronic supplementary material.

Supplementary Material 1

## Data Availability

No datasets were generated or analysed during the current study.
