# Supplementary material for: Sex differences in the relationship between pain and autonomic outflow during a cold pressor test
Source: Biol Sex Differ. 2025 Aug 6;16:60. doi: 10.1186/s13293-025-00743-2 (PMC12326594; doi:10.1186/s13293-025-00743-2)
Supplement: Supplementary file 1 — Supplementary Material 1 [file 13293_2025_743_MOESM1_ESM.docx]

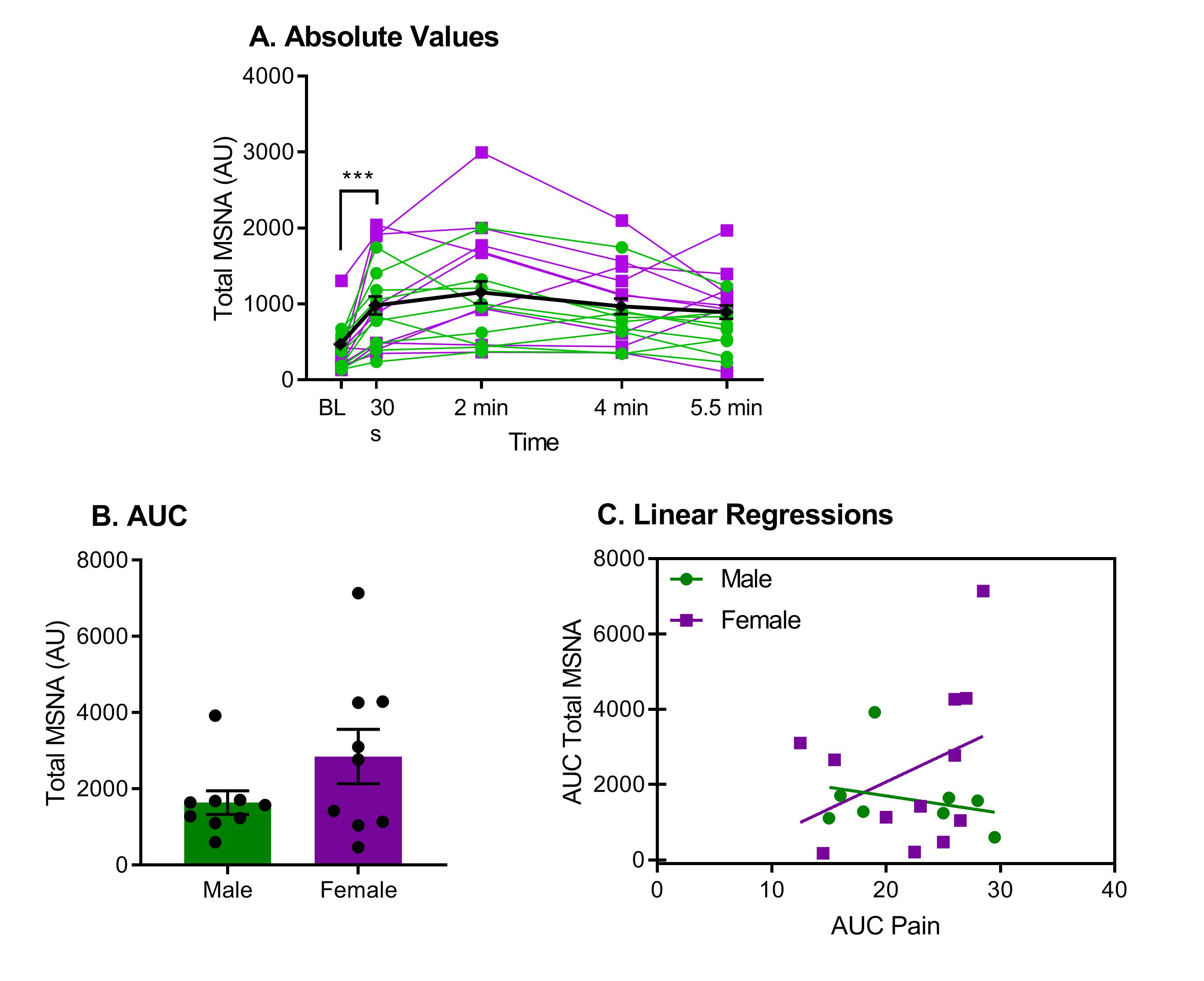


Supplementary Fig. 1. Total MSNA (MSNA burst frequency x MSNA burst amplitude) in study participants. A) Absolute values of all participants. Black symbols indicate mean ± SEM; female participants in purple and male participants in green. B) Area under the curve (AUC) of male and female participants. C) Relationship between pain ratings (AUC over the entire 5.5-min cold-pressor test) and total MSNA in male and female participants. ****p*<0.001 as indicated (means).
